# Associations of blood pressure in the third trimester and risk of venous thromboembolism postpartum

**DOI:** 10.1002/mco2.619

**Published:** 2024-06-27

**Authors:** Qian Li, Hongfei Wang, Huafang Wang, Jun Deng, Zhipeng Cheng, Fengjuan Fan, Wenyi Lin, Ruiqi Zhu, Shi Chen, Jinrong Guo, Yuxiong Weng, Liang V. Tang, Yu Hu

**Affiliations:** ^1^ Institute of Hematology Union Hospital, Tongji Medical College, Huazhong University of Science and Technology Wuhan Hubei China; ^2^ Institute of Hematology Key Lab of Molecular Biological Targeted Therapies of the Ministry of Education, Union Hospital, Tongji Medical College, Huazhong University of Science and Technology Wuhan Hubei China; ^3^ Department of Cardiovascular Surgery Union Hospital, Tongji Medical College, Huazhong University of Science and Technology Wuhan Hubei China; ^4^ Department of Biobank Union Hospital, Tongji Medical College, Huazhong University of Science and Technology Wuhan Hubei China; ^5^ Department of Medical Records Management and Statistics Union Hospital, Tongji Medical College, Huazhong University of Science and Technology Wuhan Hubei China; ^6^ Department of Hand Surgery Union Hospital, Tongji Medical College, Huazhong University of Science and Technology Wuhan Hubei China

**Keywords:** diastolic blood pressure, postpartum, pregnant women, systolic blood pressure, venous thromboembolism

## Abstract

Studies on the associations of blood pressure (BP) and the risk of venous thromboembolism (VTE) had been performed neither among pregnant women nor in Chinese population. This study included participants of pregnant women from a retrospective multicenter cohort, between May 2020 and April 2023. Systolic BP (SBP) and diastolic BP (DBP) of the participants were measured in the third trimester. The incidences of VTE (including deep venous thrombosis and/or pulmonary embolism) at 42 days postpartum were followed. With regards to SBP, pregnant women in the Q1 (≤114 mmHg), Q2 (115–122 mmHg), and Q4 group (≥131 mmHg) had increased risk of VTE than those in Q3 group (123–130 mmHg), with ORs 4.48 [1.69, 11.85], 3.52 [1.30, 9.59], and 3.17 [1.12, 8.99], respectively. Compared with pregnant women with the Q4 of DBP (≥85 mmHg), women of Q1 (≤71 mmHg) were found to have elevated risk of VTE (OR 2.73 [1.25, 5.96]). A one standard deviation decrease of DBP (9 mmHg) was related with 37% elevated risk of VTE (OR 1.37 [1.05, 1.79]). This study demonstrated a U‐shaped association of SBP in the third trimester and VTE postpartum and inverse association of DBP in the third trimester and VTE postpartum.

## Graphical abstract

Adjusted log (OR) (solid line) and 95% CIs (dashed line) for VTE postpartum by SBP (A) and DBP (B) in the third trimester

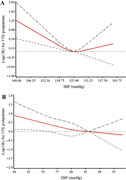

## 1 INTRODUCTION

The incidence of venous thromboembolism (VTE) is increasing year by year worldwide, which attracts more attention of clinicians and scholars.^1^, ^2^ VTE, including deep venous thrombosis (DVT) and pulmonary embolism (PE), is a major cause of maternal mortality and morbidity.^3^, ^4^ Our and other studies have identified risk factors for VTE among pregnant women, including characteristics of the mothers (e.g., body mass index (BMI), age, multiple pregnancy),^5^ and characteristics of obstetric (e.g., cesarean delivery, preterm delivery, preeclampsia),^5^, ^6^, ^7^ and nutrition status of the mothers (e.g., serum alkaline phosphatase).^8^ Abnormal blood pressure (BP) was also found to be related to inflammation and abnormal expression of the adhesion molecules, which may lead to VTE.^9^, ^10^ BP can be ameliorated with management, appropriate therapy, and/or lifestyle changes.^11^ Whether BP is a high‐risk factor of VTE and whether management of BP can reduce the risk of VTE is a public health problem that clinicians and scholars worldwide pay close attention to.

BP measurements are routinely obtained among community and pregnant women for the diagnosis and treatment of hypertensive disorders.^12^ The pattern of arterial function of pregnant women was different from nonpregnant women.^11^ There was study suggesting that arterial pressures (including systolic blood pressure [SBP] and diastolic blood pressure [DBP]) reached a nadir in the second trimester, and followed by an elevation in the third trimester and after delivery.^13^ There were also other studies demonstrating a progressive increase of BP across pregnancy.^14^, ^15^ Therefore, the dynamic changes of BP across pregnancy are unknown and the relationships of BP and VTE postpartum remain unclear.

Raised BP was found to be a major risk factor for cardiovascular disease.^16^, ^17^ Previous studies had investigated the associations of BP and the risk of VTE, but reached conflicting conclusions. A meta‐analysis including nine prospective studies reported that SBP was negatively associated with the occurrences of VTE, while DBP was not.^18^ Studies from populations in the UK,^19^ the USA,^20^ and the Netherlands^20^ found negative associations between SBP and/or DBP and VTE, but studies from Japanese^21^ and Danish^22^ populations found the opposite. An analysis of two separate cohorts of the Emerging Risk Factors Collaboration (ERFC) and the UK Biobank found inconsistent associations between BP and VTE.^23^ A cross‐sectional retrospective observational study in the USA found BP was a helpful predictor for in‐hospital VTE among patients with COVID‐19.^24^ Taken together, the associations of BP and VTE remain controversial. So far, prior study has investigated the relationships of BP and VTE neither among pregnant women nor in Chinese population.

Therefore, for the first time, based on a multicenter cohort in China, the purpose of the current study was to figure out the links of BP in the third trimester and the risk of VTE postpartum, and to provide data support for establishing optimal reference ranges of BP in the third trimester in the prevention of VTE postpartum.

## 2 RESULTS

### 2.1 Enrollment of the study population

**FIGURE 1:**
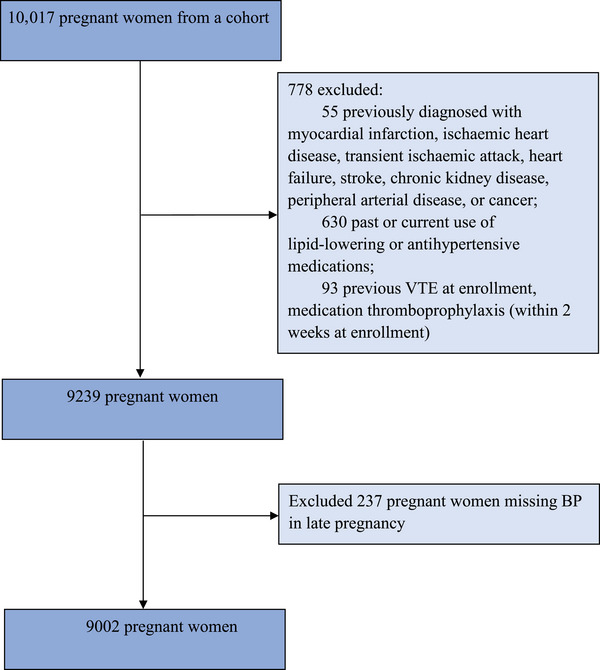
Flow diagram of this study. BP, blood pressure; VTE, venous thromboembolism.

Between May 2020 and April 2023, 10,017 pregnant women were enrolled. After exclusion, 9002 pregnant women who met the inclusion criteria of this study were included. The flow chart of the study is shown in Figure 1.

### 2.2 Characteristics of the study population

The characteristics of the pregnant women according to quartiles of SBP and DBP are described in Tables 1 and S1, respectively. In this study, a total of 58 (0.64%, 58 out of 9002) pregnant women had VTE postpartum. Among the cases, 86.2% (50 out of 58) were diagnosed with DVT only, 6.9% (four out of 58) with PE only, and 6.9% (four out of 58) with DVT and PE.

**TABLE 1:** Characteristics of the pregnant women according to quartiles of SBP (*n* = 9002).

		SBP (mmHg)				
	Total	Q1 (Lowest) (≤114)	Q2 (115–122)	Q3 (123–130)	Q4 (Highest) (≥131)	*p* Value
*n*	9002	2445	2344	2337	1876	
Maternal age (years)	31.0 (28.0, 33.0)	31.0 (28.0, 33.0)	31.0 (28.0, 33.0)	31.0 (28.0, 33.0)	31.0 (28.0, 34.0)	0.119
IVF pregnancy (%)	834 (8.3)	160 (6.5)	184 (7.8)	181 (7.7)	160 (8.5)	0.091
Multiple pregnancy (%)	116 (1.3)	25 (1.0)	31 (1.3)	39 (1.7)	21 (1.1)	0.216
Primipara (%)	5375 (59.7)	1400 (57.3)	1411 (60.2)	1409 (60.3)	1155 (61.6)	0.025^*^
Family history of hypertension	157 (1.7)	34 (1.4)	36 (1.5)	49 (2.1)	38 (2.0)	0.176
Smoking habit (%)	7 (0.1)	2 (0.1)	1 (0.04)	2 (0.1)	2 (0.1)	0.897
Alcohol drinking habit (%)	8 (0.1)	1 (0.04)	1 (0.04)	4 (0.2)	2 (0.1)	0.385
GDM (%)	2012 (22.4)	546 (22.3)	513 (21.9)	521 (22.3)	432 (23.0)	0.851
Preeclampsia (%)	68 (0.8)	2 (0.1)	4 (0.2)	8 (0.3)	54 (2.9)	<0.001^*^
Preterm (%)	790 (8.8)	227 (9.3)	203 (8.7)	205 (8.8)	155 (8.3)	0.695
VTE (%)	58 (0.64%)	23 (0.94%)	17 (0.73%)	5 (0.21%)	13 (0.69%)	0.015^*^

Abbreviations: BMI, body mass index; GDM, gestational diabetes mellitus; IVF, in vitro fertilization; SBP, systolic blood pressure.

* *p* < 0.05.

The participants’ median age at enrollment was 31.0 (28.0, 33.0) years old. A sum of 834 (8.3%) pregnant women had IVF pregnancy, 116 (1.3%) had multiple pregnancy, and 5375 (59.7%) were primipara, respectively. The characteristics of the participants included and excluded (due to lost information of BP or other reasons) were similar (Table S2).

### 2.3 BP in the third trimester

**FIGURE 2:**
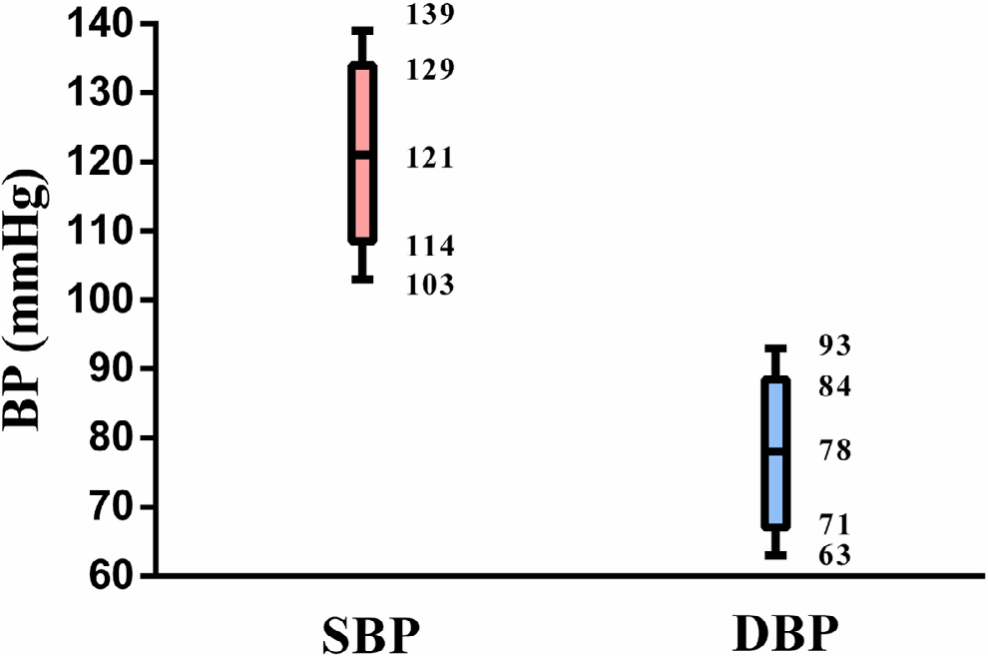
Box plot of BP (SBP and DBP). BP, blood pressure; DBP, diastolic blood pressure; SBP, systolic blood pressure. The upper edge and the lower edge of the box indicate the 75th percentile and 25th percentile, respectively. The horizontal bar in the middle of each box plot represents of the median value. The upper horizontal line and the lower horizontal line of the box plot indicate the 95th percentile and 5th percentile, respectively.

The measured values of SBP and DBP were normally distributed. As shown in the box plot (Figure 2), the median (interquartile range [IQR]) values of SBP and DBP were 121 [114, 129] mmHg and 78 [71, 84] mmHg, respectively.

### 2.4 Associations of SBP and VTE

Associations of quartiles of SBP and VTE are shown in Table 2. In the unadjusted model, compared with pregnant women with the Q3 of SBP (123–130 mmHg), pregnant women in the group of Q1 (≤114 mmHg), Q2 (115–122 mmHg), and Q4 (≥131 mmHg) had increased risk of VTE, with ORs 4.43 [1.68, 11.67], 3.41 [1.26, 9.25], and 3.26 [1.16, 9.15], respectively. In the model 1, compared with pregnant women with the Q3 of SBP, pregnant women in the group of Q1, Q2, and Q4 had increased risk of VTE, with ORs 4.37 [1.67, 11.51], 3.46 [1.28, 9.41], and 3.24 [1.15, 9.12], respectively. In the model 2, after adjustment, compared with pregnant women with the Q3 of SBP, pregnant women in the group of Q1, Q2, and Q4 had increased risk of VTE, with ORs 4.48 [1.69, 11.85], 3.52 [1.30, 9.59], and 3.17 [1.12, 8.99], respectively. Sensitivity analyses among pregnant women without family history of hypertension (Table S3) and preeclampsia (Table S4) observed consistent findings. The results also revealed a U‐shaped association of SBP and VTE postpartum and inverse association of DBP and VTE postpartum.

**TABLE 2:** Adjusted ORs and 95% CIs for SBP and VTE risk (*n* = 9002).

	SBP (mmHg)			
	Q1 (Lowest) (≤114)	Q2 (115–122)	Q3 (123–130)	Q4 (Highest) (≥131)
No. of VTE (%)	23 (0.94%)	17 (0.73%)	5 (0.21%)	13 (0.69%)
Unadjusted	4.43 (1.68, 11.67)	3.41 (1.26, 9.25)	1.00	3.26 (1.16, 9.15)
Model 1	4.37 (1.67, 11.51)	3.46 (1.28, 9.41)	1.00	3.24 (1.15, 9.12)
Model 2	4.48 (1.69, 11.85)	3.52 (1.30, 9.59)	1.00	3.17 (1.12, 8.99)

Abbreviations: OR, odds ratio; SBP, systolic blood pressure; VTE, venous thromboembolism.

Model 1 adjusted for maternal age, in vitro fertilization pregnancy, multiple pregnancy, primipara, and family history of hypertension.

Model 2 adjusted for alcohol drinking habit, smoking habit, BMI at enrollment, history of diabetes, gestational diabetes mellitus, preeclampsia, preterm, postpartum hemorrhage, delivery mode, medication thromboprophylaxis after enrollment, and covariates included in model 1.

### 2.5 Associations of DBP and VTE

Associations of quartiles of DBP and VTE are presented in Table 3. In model 2, after adjustment, higher VTE risk was observed in pregnant women with the Q1 of DBP (≤71 mmHg) compared with those with the Q4 (≥85 mmHg) with OR 2.73 [1.25, 5.96]. A one SD (standard deviation) decrease of DBP (9 mmHg) was related with increased risk of VTE with OR 1.34 [1.03, 1.75], 1.33 [1.02, 1.74], and 1.37 [1.05, 1.79] in the unadjusted model, model 1, and model 2, respectively. Sensitivity analyses among pregnant women without family history of hypertension (Table S5) and preeclampsia (Table S6) also found the inverse links of DBP and VTE.

**TABLE 3:** Adjusted ORs and 95% CIs for DBP and VTE risk (*n* = 9002).

	DBP (mmHg)					
	Q1 (Lowest) (≤71)	Q2 (72–77)	Q3 (78–84)	Q4 (Highest) (≥85)	Per‐SD decrease of DBP	*P*_trend_
No. of VTE (%)	26 (1.13%)	10 (0.47%)	13 (0.52%)	9 (0.43%)		
Unadjusted	2.63 (1.23, 5.62)	1.08 (0.44, 2.65)	1.20 (0.51, 2.81)	1.00	1.34 (1.03, 1.75)	0.012
Model 1	2.57 (1.20, 5.51)	1.04 (0.42, 2.57)	1.18 (0.50, 2.77)	1.00	1.33 (1.02, 1.74)	0.013
Model 2	2.73 (1.25, 5.96)	1.08 (0.43, 2.71)	1.26 (0.53, 3.01)	1.00	1.37 (1.05, 1.79)	0.011

Abbreviations: DBP, diastolic blood pressure; OR, odds ratio; SD, standard deviation; VTE, venous thromboembolism.

Model 1 adjusted for maternal age, in vitro fertilization pregnancy, multiple pregnancy, primipara, and family history of hypertension.

Model 2 adjusted for alcohol drinking habit, smoking habit, BMI at enrollment, history of diabetes, gestational diabetes mellitus, preeclampsia, preterm, postpartum hemorrhage, delivery mode, medication thromboprophylaxis after enrollment, and covariates included in model 1.

## 3 DISCUSSION

This is the first study on relationships of BP and VTE in pregnant women worldwide, and the first to be conducted in China. This study found a U‐shaped association between SBP in the third trimester and VTE postpartum, and inverse association between DBP in the third trimester and VTE postpartum. Both low and high SBP were related to higher odds of newly diagnosed VTE postpartum. Low DBP was associated with elevated risk of VTE postpartum. Restricting the analyses among pregnant women without family history of hypertension and preeclampsia, the association of SBP/DBP and VTE remained robust.

Prior to this study, association of BP and VTE was investigated among population of inhabitants,^23^, ^25^, ^26^ patients,^24^ and advanced lung cancer.^21^ However, the results were not consistent. Similar to our results, a cross‐sectional retrospective observational study by Lee et al.^24^ suggested that BP was a helpful indicator of in‑hospital VTE in COVID‑19 patients. A meta‐analysis including 9 prospective studies observed an inverse association for SBP and VTE (160 vs. 110 mmHg, HR = 0.79 [0.68, 0.92]), but not for DBP.^18^ Mendelian randomization analyses^19^, ^26^ showed inverse associations of SBP and VTE. The Age and Thrombosis, Acquired and Genetic risk factors in the elderly (AT‐AGE) study^20^ and the second Nord‐Trøndelag Health Study (HUNT 2)^25^ found SBP and DBP were inversely correlated to VTE among elderly (age ≥70 years) and adult residents, respectively.

Oppositely, the Copenhagen City Heart Study (from Denmark) found DBP was significantly associated with VTE (100 vs. 80 mmHg, HR, 1.34 [1.08, 1.66]).^22^ The Rising‐VTE/NEJ037 Study (from Japan) reported that DBP ≥70 mmHg was a risk factor for VTE (OR, 1.931 [1.122, 3.325]).^21^ The “study of men born in 1913”^27^ and the Atherosclerosis Risk in Communities study (ARIC) (from the USA)^28^, ^29^ implied that BP was not related to VTE risk among inhabitants. Gregson et al.^23^ even found inconsistent associations of BP with VTE in UK Biobank and the ERFC in the same paper. Therefore, the association between BP and VTE is very complex, and the conclusions vary from study to study. We hypothesized that this might be related to the variance in the BP status, age, sex, disease status, country or region, genetics characteristics, nutrition, lifestyle of the study population, study designs, covariates considered, and the definitions of the outcomes. The current study is the first among pregnant population, and the first in Chinese population, we should be cautious when extrapolating the conclusions of this study to other populations.

Family history of hypertension and preeclampsia appeared to be nonmodifiable risk factors for hypertensive disorders in pregnancy which related to VTE risk.^30^, ^31^ Women with preeclampsia have been shown to have fivefold higher risk of VTE during the periconceptional period than the general population.^7^ Therefore, pregnant women with family history of hypertension and preeclampsia were excluded from the sensitivity analyses in this study. Previous study hypothesized that the inverse links of BP and VTE risk might be ascribed to medication thromboprophylaxis.^18^ In the adjusted models, these covariates of medication thromboprophylaxis, family history of hypertension and preeclampsia were adjusted, and the results remained robust, suggesting that the relationships of BP and VTE were independent of these covariates.

Compared with hereditary risk factors, the risk factor of abnormal BP can be ameliorated with management, appropriate therapy and/or lifestyle changes.^11^ In clinical practice, BP needs to be continuously monitored and controlled from the time of pregnancy to delivery.^12^ Abnormal BP may lead to a variety of adverse maternal and fetal outcomes.^32^, ^33^, ^34^, ^35^ Most previous studies had found that BP of pregnant women increased from early pregnancy, decreased during the middle pregnancy (due to the development of the low‐resistance uteroplacental vascular system^36^ and the modification of the renin–angiotensin–aldosterone system),^37^ and then continued to increase until delivery.^13^ However, there were also studies which found that BP in pregnant women increased progressively from early pregnancy to delivery.^14^, ^15^ According to the existing guidelines, gestational hypertension is usually diagnosed with a SBP ≥140 mmHg and/or a DBP ≥90 mmHg after 20 weeks of gestation.^38^, ^39^ In clinical practice, lowering BP was effective in reducing cardiovascular disease risk.^40^ In combination with the results from this study, BP in the third trimester is not the lower the better, even in the normal ranges. High SBP is a risk factor for VTE postpartum. Participants with high SBP need nondrug therapies (e.g., changes in diet and lifestyle habits) and/or antihypertensive therapy. Appropriate physical activity in the third trimester appeared to had significant benefits for healthy (safe for most healthy pregnant women) and hypertensive pregnant women (under the guidance of the doctors) in terms of hemodynamic status and arterial functions.^11^ More studies on useful clinical practices of BP management in the third trimester should be conducted in the future.

The mechanisms of the U‐shaped or inverse association of SBP/DBP and VTE found in this study remain unclear. According to Virchow's triad, VTE was formed by the imbalance of the opposing coagulative and fibrinolytic systems. The three main causes for VTE includes changes of vessel wall damage, hypercoagulability, and circulatory stasis (stasis or low flow, specifically low SBP/DBP).^41^ The associations of high SBP with VTE may be explained, in part, by hypertension^42^, ^43^ or arteriosclerosis^44^ caused under the circumstances of high SBP, which may lead to higher VTE risk. Under the circumstances of low SBP/DBP, it reduces flow of oxygenated blood in veins, leading the endothelium to hypoxemia. Hypoxia, metabolic stress, and inflammatory cytokines could activate endothelial cells. Hypoxemic endothelium is associated with inflammation and abnormal expression of the adhesion molecules,^9^, ^10^ which may trigger the coagulation cascade.^41^, ^45^ These may partly explain the relationships of low SBP/DBP and increased VTE risk observed in this study. It is also possible that nutrients such as manganese is involved in the mechanisms. Prior study found that both SBP and DBP were associated with manganese levels in the body,^46^ whereas higher levels of manganese‐dependent enzyme were correlated with increased antioxidant activity in oxidative stress conditions,^47^ possibly leading to elevated risk of VTE. More experimental studies are warranted to underline the potential mechanisms.

Our study has several strengths that worth mentioning. First, this is the first study on associations of BP and VTE which is conducted in China, and the first among pregnant women worldwide. This study provides a scientific basis for establishing the optimal reference ranges of BP in the third trimester. Second, this study had the ability to exclude pregnant women with past or current use of lipid‐lowering or antihypertensive medications which may affect the development of VTE. This study also adjusted for covariate of medication thromboprophylaxis after enrollment, which purified the current results. Third, the validation of VTE events is standardized and thorough, all the diagnoses were confirmed by two independent researchers, adjudicated by another researcher if any difference exists, which made the data of diagnoses of VTE and other diseases reliable. The covariates (including social demographic, history of reproduction, family history of disease, history of disease, life style, delivery information, and medication use) are relatively comprehensive which allows for adjustments and sensitivity analyses.

Inevitably, this study has limitations. First, this is a retrospective cohort. The retrospective study design was not able to establish a causal link of BP and VTE, although previous Mendelian randomization studies have provided sufficient evidence of a causal link of BP and VTE.^19^, ^26^ Second, participants with hereditary risk factors had elevated risk of VTE than controls.^48^ Genetic factors were not able to be taken into consideration in this study. It remains unknown whether or to what extent genetic factors affected the associations of BP and VTE in this study. Third, additional confounders (e.g., intrauterine growth restriction, immobilization, and coagulation function) may still exist, though this study has included potential confounding comprehensively. Fourth, the representativeness of the study population and the sample size need to be expanded, and the conclusions in this study are unknown in terms of the stability of the larger sample size, as well as the reliability of the generalization to other populations. Therefore, the results of this study should be interpreted with caution.

In conclusion, this is the first study, to our knowledge, demonstrating a U‐shaped association of SBP in the third trimester and VTE postpartum, and inverse associations of DBP in the third trimester and VTE postpartum, suggesting that BP in the third trimester could be a useful predictor for VTE postpartum. The current study developed a scientific basis for establishing the optimal reference ranges of BP in the third trimester, providing important public health implications of BP management in preventing VTE. More prospective cohort studies and mechanism researches are warranted in the future.

## 4 METHODS

This study followed the Strengthening the Reporting of Observational Studies in Epidemiology reporting guideline for cohort studies.

### 4.1 Study design and population

The participants came from a retrospective multicenter cohort of 9002 pregnant women who delivered in three designated sites in Union Hospital, Tongji Medical College, Huazhong University of Science and Technology, Wuhan, China, including the Main Campus, Che‐gu Campus, and Jin Yin‐hu Campus, between May 2020 and April 2023. The participants were enrolled in the third trimester in the hospitals, and the health status of the participants at delivery and at 42 days postpartum were followed through the medical records. This study was approved by the ethics committee of Tongji Medical College affiliated with Huazhong University of Science and Technology (no: [2015] S014). Informed consent was not required since the information was retrieved through the medical records retrospectively.

This study excluded participants (1) previously diagnosed with myocardial infarction, ischemic heart disease, transient ischemic attack, heart failure, stroke, chronic kidney disease, peripheral arterial disease, or cancer (*n* = 55); (2) past or current use of lipid‐lowering or antihypertensive medications (*n* = 630); (3) previous VTE, medication thromboprophylaxis (within two weeks at enrollment) (*n* = 93); (4) missing BP in the third trimester (*n* = 237).

### 4.2 Data collection

Information of social demography, BP, and disease diagnoses was collected from the medical records. Disease diagnoses were made according to the validated International Classification of Diseases, 10th.^49^ All the diagnoses of VTE were checked by Qian Li and Hongfei Wang, the interpretation was discussed with Liang V Tang if any difference exists.

### 4.3 BP measurement

The pregnant women were asked to measurement BP at enrollment by the trained medical workers in the designated hospitals. After 5‐min rest, the first measurement of BP began. After the first measurement, with 1‐min rest, the second measurement of BP began. The BP was recorded according to the average values of the two measurements. In this study, the participants were categorized according to the quartiles of BP.

### 4.4 Diagnosis of VTE

Participants who had new‐onset VTE (DVT and/or PE) within 42 days after delivery were defined as VTE cases. Diagnoses of DVT and PE were described in our previous study detailly.^8^

### 4.5 Definition of the covariates

Covariates of maternal age, in vitro fertilization pregnancy, multiple pregnancy, primipara, family history of hypertension, alcohol drinking habit, smoking habit, BMI at enrollment, history of diabetes, gestational diabetes mellitus, preeclampsia, preterm, postpartum hemorrhage, delivery mode, and medication thromboprophylaxis after enrollment were adjusted. GDM was diagnosed according to the recommendation by the International Association of the Diabetes and Pregnancy Study Groups.^50^ Preterm was defined as babies born alive before 37 weeks of pregnancy.^51^

### 4.6 Statistical analysis

Median (IQR) and *n* (%) were used to show continuous and categorical variables in this study. Logistic regression models were applied to explore the odds ratios (ORs) and 95% confidence intervals (CIs) for VTE postpartum according to quartiles of SBP (reference group Q3) and DBP (reference group Q4) in the third trimester. Model 1 adjusted for maternal age, in vitro fertilization pregnancy, multiple pregnancy, primipara, and family history of hypertension. Model 2 adjusted for alcohol drinking habit, smoking habit, BMI at enrollment, history of diabetes, gestational diabetes mellitus, preeclampsia, preterm, postpartum hemorrhage, delivery mode, medication thromboprophylaxis after enrollment, and covariates included in model 1. Logistic regression models were used in investigating the associations of per‐SD decrease of DBP and VTE postpartum. To test the robustness of the results, sensitivity analyses were conducted among participants without family history of hypertension and preeclampsia. The analyses were performed with SPSS version 21.0. Two‐sided *p* value < 0.05 was considered as statistically significant.

## AUTHOR CONTRIBUTIONS

Yu Hu, Liang V. Tang, Qian Li, and Hongfei Wang concepted this study. Qian Li, Liang V. Tang, Hongfei Wang, Huafang Wang, Jun Deng, Zhipeng Cheng, Fengjuan Fan, Wenyi Lin, Ruiqi Zhu, Shi Chen, Jinrong Guo, and Yuxiong Weng analyzed the data. Qian Li, Yu Hu, Liang V. Tang, and Hongfei Wang drafted the manuscript. All authors had full access to the data and verified the results in this study. All authors revised the manuscript and gave final approval for the version to be published.

## CONFLICT OF INTEREST STATEMENT

All authors declare no conflict of interest.

## ETHICS STATEMENT

This study was approved by the ethics committee of Tongji Medical College affiliated with Huazhong University of Science and Technology (no: [2015] S014). Informed consent was not required since the information was retrieved through the medical records retrospectively.

## Supporting information

Supporting Information

## Data Availability

All data generated or analyzed during this study are included in this published article (and its Supporting Information files).
