# Supplementary material for: Associations of blood pressure in the third trimester and risk of venous thromboembolism postpartum
Source: MedComm (2020). 2024 Jun 27;5(7):e619. doi: 10.1002/mco2.619 (PMC11208741; doi:10.1002/mco2.619)
Supplement: Supplementary file 1 — Supporting Information [file MCO2-5-e619-s001.docx]

**Title**: Associations of blood pressure in the third trimester and risk of venous thromboembolism postpartum

**A running title**: Blood pressure and VTE postpartum

**Authors:** Qian Li^1,2,^#, PhD, Hongfei Wang^3^,# PhD, Huafang Wang^1,2^, PhD, Jun Deng^1,2^, PhD, Zhipeng Cheng^1,2^, PhD, Fengjuan Fan^1,2^, PhD, Wenyi Lin^1,2^, PhD, Ruiqi Zhu^1,2^, PhD, Shi Chen^4^, MPH, Jinrong Guo^5^, MPH, Yuxiong Weng^6^, PhD, Liang V Tang^1,2^,* PhD, Yu Hu^1,2,^*, PhD

#Contributed equally

*Contributed equally

**Affiliations:**

^1^Institute of Hematology, Union Hospital, Tongji Medical College, Huazhong University of Science and Technology, Wuhan, Hubei, China

^2^Key Lab of Molecular Biological Targeted Therapies of the Ministry of Education, Union Hospital, Tongji Medical College, Huazhong University of Science and Technology, Wuhan, Hubei, China

^3^Department of Cardiovascular Surgery, Union Hospital, Tongji Medical College, Huazhong University of Science and Technology, Wuhan, Hubei, China

^4^Department of Biobank, Union Hospital, Tongji Medical College, Huazhong University of Science and Technology, Wuhan, Hubei, China

^5^Department of Medical Records Management and Statistics, Union Hospital, Tongji Medical College, Huazhong University of Science and Technology, Wuhan, Hubei, China

^6^Department of Hand Surgery, Union Hospital, Tongji Medical College, Huazhong University of Science and Technology, Wuhan, Hubei, China

**Correspondence to:**

Yu Hu, Institute of Hematology, Union Hospital, Tongji Medical College, Huazhong University of Science and Technology, Wuhan, 430022, Hubei, China. Key Lab of Molecular Biological Targeted Therapies of the Ministry of Education, Union Hospital, Tongji Medical College, Huazhong University of Science and Technology, Wuhan, Hubei, China. Tel: +86-027-85726387. Email: [dr_huyu@126.com](mailto:dr_huyu@126.com)

Liang V Tang, Institute of Hematology, Union Hospital, Tongji Medical College, Huazhong University of Science and Technology, Wuhan, 430022, Hubei, China. Key Lab of Molecular Biological Targeted Therapies of the Ministry of Education, Union Hospital, Tongji Medical College, Huazhong University of Science and Technology, Wuhan, Hubei, China. Tel: +86-027-85726005. Email: [lancet.tang@qq.com](mailto:lancet.tang@qq.com)

**Table S1—Characteristics of the pregnant women according to quartiles of DBP (n=9002)**

|  | **Total** | **DBP (mmHg)** | | | | ***P*** |
| --- | --- | --- | --- | --- | --- | --- |
|  |  | **Q1 (Lowest)**  **(≤71)** | **Q2**  **(72-77)** | **Q3**  **(78-84)** | **Q4 (Highest)**  **(≥85)** |  |
| **n** | 9002 | 2293 | 2139 | 2499 | 2071 |  |
| **Maternal age (years)** | 31.0 (28.0, 33.0) | 31.0 (28.0, 33.0) | 31.0 (28.0, 34.0) | 31.0 (28.0, 34.0) | 31.0 (28.0, 34.0) | 0.027* |
| **IVF pregnancy (%)** | 834 (8.3) | 57 (6.8) | 150 (7.0) | 191 (7.6) | 187 (9.0) | 0.030* |
| **Multiple pregnancy (%)** | 116 (1.3) | 36 (1.6) | 27 (1.3) | 30 (1.2) | 23 (1.1) | 0.550 |
| **Primipara (%)** | 5375 (59.7) | 1295 (56.5) | 1258 (58.8) | 1528 (61.1) | 1294 (62.5) | <0.001* |
| **Family history of hypertension** | 157 (1.7) | 35 (1.5) | 37 (1.7) | 45 (1.8) | 40 (1.9) | 0.775 |
| **Smoking habit (%)** | 7 (0.1) | 4 (0.2) | 2 (0.1) | 1 (0.04) | 0 (0.0) | 0.180 |
| **Alcohol drinking habit (%)** | 8 (0.1) | 1 (0.04) | 5 (0.2) | 1 (0.04) | 1 (0.05) | 0.084 |
| **GDM (%)** | 2012 (22.4) | 492 (21.5) | 489 (22.9) | 541 (21.6) | 490 (23.7) | 0.247 |
| **Preeclampsia (%)** | 68 (0.8) | 5 (0.2) | 0 (0.0) | 7 (0.3) | 56 (2.7) | <0.001* |
| **Preterm (%)** | 790 (8.8) | 226 (9.9) | 182 (8.5) | 218 (8.7) | 164 (7.9) | 0.142 |
| **VTE (%)** | 58 (0.64%) | 26 (1.13%) | 10 (0.47%) | 13 (0.52%) | 9 (0.43%) | 0.009* |

Abbreviations: BMI: body mass index; DBP: diastolic blood pressure; GDM: gestational diabetes mellitus; IVF: *in vitro* fertilization.

**P* <0.05

**Table S2—Characteristics of the participants included and excluded from this study**

| **Characteristics** | **Total**  **(n = 10017)** | **Included**  **(n = 9002)** | **Excluded due to missing BP**  **(n = 237)** | **Excluded due to other reasons**  **(n = 778)** |
| --- | --- | --- | --- | --- |
| **Maternal age (years)** | 31.0 (28.0, 34.0) | 31.0 (28.0, 33.0) | 31.0 (28.0, 34.0) | 32.0 (29.0, 35.0) |
| **Height at enrollment (cm)** | 161.0 (158.0, 165.0) | 161.0 (158.0, 165.0) | 160.0 (157.0, 164.5) | 160.0 (158.0, 164.0) |
| **Weight at enrollment (kg)** | 68.6 (62.5, 75.0) | 68.0 (62.2, 75.0) | 66.0 (58.0, 72.0) | 72.8 (65.0, 80.0) |
| **BMI at enrollment (kg/m^2^)** | 26.3 (24.1, 28.7) | 26.2 (24.0, 28.4) | 25.5 (23.1, 28.6) | 28.3 (25.4, 31.0) |

Abbreviations: BMI: body mass index; IVF: *in vitro* fertilisation.

**Table S3—Adjusted ORs and 95% CIs for SBP and VTE risk among pregnant women without family history of hypertension (n=8845)**

|  | **SBP (mmHg)** | | | |
| --- | --- | --- | --- | --- |
|  | **Q1 (Lowest)**  **(≤114)** | **Q2**  **(115-122)** | **Q3**  **(123-130)** | **Q4 (Highest)**  **(≥131)** |
| **No. of VTE, (%)** | 23 (0.95%) | 16 (0.69%) | 5 (0.22%) | 13 (0.71%) |
| **Unadjusted** | 4.40 (1.67, 11.59) | 3.19 (1.17, 8.72) | 1.00 | 3.25 (1.16, 9.14) |
| **Model 1** | 4.35 (1.65, 11.47) | 3.25 (1.19, 8.90) | 1.00 | 3.26 (1.16, 9.16) |
| **Model 2** | 4.50 (1.70, 11.89) | 3.31 (1.21, 9.08) | 1.00 | 3.19 (1.13, 9.05) |

Abbreviations: OR: odds ratio; SBP: systolic blood pressure; VTE: venous thromboembolism.

Model 1 adjusted for maternal age, *in vitro* fertilization pregnancy, multiple pregnancy, and primipara.

Model 2 adjusted for alcohol drinking habit, smoking habit, body mass index at enrollment, history of diabetes, gestational diabetes mellitus, preeclampsia, preterm, postpartum hemorrhage, delivery mode, medication thromboprophylaxis after enrollment and covariates included in model 1.

**Table S4—Adjusted ORs and 95% CIs for SBP and VTE risk among pregnant women without preeclampsia (n=8934)**

|  | **SBP (mmHg)** | | | |
| --- | --- | --- | --- | --- |
|  | **Q1 (Lowest)**  **(≤114)** | **Q2**  **(115-122)** | **Q3**  **(123-130)** | **Q4 (Highest)**  **(≥131)** |
| **No. of VTE, (%)** | 23 (0.94%) | 17 (0.73%) | 5 (0.21%) | 12 (0.66%) |
| **Unadjusted** | 4.42 (1.68, 11.64) | 3.40 (1.25, 9.24) | 1.00 | 3.08 (1.08, 8.76) |
| **Model 1** | 4.37 (1.66, 11.52) | 3.46 (1.27, 9.40) | 1.00 | 3.06 (1.07, 8.70) |
| **Model 2** | 4.46 (1.69, 11.79) | 3.51 (1.29, 9.55) | 1.00 | 3.11 (1.09, 8.86) |

Abbreviations: OR: odds ratio; SBP: systolic blood pressure; VTE: venous thromboembolism.

Model 1 adjusted for maternal age, *in vitro* fertilization pregnancy, multiple pregnancy, primipara and family history of hypertension.

Model 2 adjusted for alcohol drinking habit, smoking habit, body mass index at enrollment, history of diabetes, gestational diabetes mellitus, preterm, postpartum hemorrhage, delivery mode, medication thromboprophylaxis after enrollment and covariates included in model 1.

**Table S5—Adjusted ORs and 95% CIs for DBP and VTE risk among pregnant women without family history of hypertension (n=8845)**

|  | **DBP (mmHg)** | | | |  |  |
| --- | --- | --- | --- | --- | --- | --- |
|  | **Q1 (Lowest)**  **(≤71)** | **Q2**  **(72-77)** | **Q3**  **(78-84)** | **Q4 (Highest)**  **(≥85)** | **Per-SD decrease of DBP** | ***P*_trend_** |
| **No. of VTE, (%)** | 26 (1.15%) | 10 (0.48%) | 12 (0.49%) | 9 (0.44%) |  |  |
| **Unadjusted** | 2.62 (1.22, 5.60) | 1.07 (0.44, 2.65) | 1.10 (0.46, 2.63) | 1.00 | 1.36 (1.04, 1.78) | 0.010 |
| **Model 1** | 2.55 (1.19, 5.47) | 1.03 (0.42, 2.54) | 1.08 (0.46, 2.58) | 1.00 | 1.35 (1.03, 1.77) | 0.011 |
| **Model 2** | 2.72 (1.24, 5.94) | 1.07 (0.43, 2.69) | 1.16 (0.48, 2.82) | 1.00 | 1.39 (1.06, 1.83) | 0.008 |

Abbreviations: DBP: diastolic blood pressure; OR: odds ratio; SD: standard deviation; VTE: venous thromboembolism.

Model 1 adjusted for maternal age, *in vitro* fertilization pregnancy, multiple pregnancy, and primipara.

Model 2 adjusted for alcohol drinking habit, smoking habit, body mass index at enrollment, history of diabetes, gestational diabetes mellitus, preeclampsia, preterm, postpartum hemorrhage, delivery mode, medication thromboprophylaxis after enrollment and covariates included in model 1.

**Table S6—Adjusted ORs and 95% CIs for DBP and VTE risk among pregnant women without preeclampsia (n=8934)**

|  | **DBP (mmHg)** | | | |  |  |
| --- | --- | --- | --- | --- | --- | --- |
|  | **Q1 (Lowest)**  **(≤71)** | **Q2**  **(72-77)** | **Q3**  **(78-84)** | **Q4 (Highest)**  **(≥85)** | **Per-SD decrease of DBP** | ***P*_trend_** |
| **No. of VTE, (%)** | 26 (1.14%) | 10 (0.47%) | 13 (0.52%) | 8 (0.40%) |  |  |
| **Unadjusted** | 2.88 (1.30, 6.38) | 1.18 (0.46, 2.99) | 1.32 (0.54, 3.18) | 1.00 | 1.40 (1.07, 1.84) | 0.009 |
| **Model 1** | 2.84 (1.28, 6.30) | 1.14 (0.45, 2.90) | 1.30 (0.54, 3.15) | 1.00 | 1.40 (1.06, 1.84) | 0.010 |
| **Model 2** | 2.85 (1.28, 6.34) | 1.12 (0.44, 2.84) | 1.31 (0.54, 3.18) | 1.00 | 1.40 (1.06, 1.85) | 0.009 |

Abbreviations: DBP: diastolic blood pressure; OR: odds ratio; SD: standard deviation; VTE: venous thromboembolism.

Model 1 adjusted for maternal age, *in vitro* fertilization pregnancy, multiple pregnancy, primipara and family history of hypertension.

Model 2 adjusted for alcohol drinking habit, smoking habit, body mass index at enrollment, history of diabetes, gestational diabetes mellitus, preterm, postpartum hemorrhage, delivery mode, medication thromboprophylaxis after enrollment and covariates included in model 1.
